# The effect of yoga on women with secondary arm lymphoedema from breast cancer treatment

**DOI:** 10.1186/1472-6882-12-66

**Published:** 2012-05-28

**Authors:** Annette Loudon, Tony Barnett, Neil Piller, Maarten A Immink, Denis Visentin, Andrew D Williams

**Affiliations:** 1 University Department of Rural Health, University of Tasmania, Launceston, TAS, 7250, Australia; 2 School of Medicine, Flinders University, Adelaide, South Australia, 5042, Australia; 3 School of Health Sciences, University of South Australia, Adelaide, South Australia, 5000, Australia; 4 School of Human Life Sciences, University of Tasmania, Launceston, TAS, 7250, Australia

**Keywords:** Yoga, Arm lymphoedema, Symptoms, Quality of life, Randomised control trial

## Abstract

**Background:**

Women who develop secondary arm lymphoedema subsequent to treatment associated with breast cancer require life-long management for a range of symptoms including arm swelling, heaviness, tightness in the arm and sometimes the chest, upper body impairment and changes to a range of parameters relating to quality of life. While exercise under controlled conditions has had positive outcomes, the impact of yoga has not been investigated. The aim of this study is to determine the effectiveness of yoga in the physical and psycho-social domains, in the hope that women can be offered another safe, holistic modality to help control many, if not all, of the effects of secondary arm lymphoedema.

**Methods and design:**

A randomised controlled pilot trial will be conducted in Hobart and Launceston with a total of 40 women receiving either yoga intervention or current best practice care. Intervention will consist of eight weeks of a weekly teacher-led yoga class with a home-based daily yoga practice delivered by DVD. Primary outcome measures will be the effects of yoga on lymphoedema and its associated symptoms and quality of life. Secondary outcome measures will be range of motion of the arm and thoracic spine, shoulder strength, and weekly and daily physical activity. Primary and secondary outcomes will be measured at baseline, weeks four, eight and a four week follow up at week twelve. Range of motion of the spine, in a self-nominated group, will be measured at baseline, weeks eight and twelve. A further outcome will be the women’s perceptions of the yoga collected by interview at week eight.

**Discussion:**

The results of this trial will provide information on the safety and effectiveness of yoga for women with secondary arm lymphoedema from breast cancer treatment. It will also inform methodology for future, larger trials.

**Trial registration:**

ACTRN12611000202965

## Background

Breast cancer- related lymphoedema (BCRL) is a result of disruption to the lymphatic transport system as a result of a number of procedures associated with the treatment of breast cancer [1]. BCRL is associated with chronic or recurring swelling of the affected area and underlain by cellular and tissue changes, making the area more prone to infections. High risk indicators for lymphoedema onset are mastectomy, radiation to the axilla, axillary dissection and removal of a high number of lymph nodes [2]. In spite of recent changes to surgical and radiological procedures for breast cancer, at least 20% of women are still affected [3]. As breast cancer incidence continues to rise [4], a significant number of women will continue to get secondary lymphoedema. While the arm is the most common site, the chest, upper back or breast are also often involved. As these areas are difficult to measure, current incidence rates are likely to be significantly underestimated.

The loss of upper body function can be higher for women with BCRL than for those treated for breast cancer without lymphoedema [5]. Physical activity can be reduced not only due to the swelling, but also as a consequence of the changed sensations [6], pain [7] and fatigue [8], as well as women’s fear of exacerbating the symptoms of lymphoedema [9]. Women commonly report stiffness and impairment in the range of motion of their affected shoulder and limitations in various daily activities that depend on carrying, repetitive actions or having their arm in the same position for a prolonged period [10]. Biomechanics of the thoracic-shoulder area can be compromised [11,12], which can create further upper body impairment. Additionally, women experience feelings of abandonment and isolation, compounded by uncertainty and poor body image [13,14], which can lead to a lowered quality of life in physical, social, personal and emotional domains [15].

BCRL requires early recognition, targeted treatment and self-management for life to reduce the risk of infection and prevent the condition worsening. Treatment may include complex lymphoedema therapy involving manual lymphatic drainage (MLD) with compression bandaging as required, and daily self-management, which focuses on skin care, self-massage, specific exercises with deep breathing and wearing a compression sleeve [16,17]. Motivation and compliance with self-care are difficult to maintain [18] due to treatment fatigue and a feeling of frustration that the limb is not getting significantly better.

Participation in exercise is increasingly recommended as another option for women with BCRL to improve their physical and mental health [19,20]. Guidelines state that supervised exercise with adequate warm-up and cool-down, and gradual progression of intensity or difficulty, may be followed by women with BCRL [21]. This is based on positive results from large randomised controlled trials of resistance training [22,23] and from smaller trials covering a variety of other exercise modalities [24] including combined aerobic and resistance exercise [25], tai-chi [26], gentle exercise with relaxation [27] and aquatic exercise [28]. Importantly, these trials have reported no exacerbation of lymphoedema and an improvement in physical fitness, upper body function and quality of life.

Currently, there is no published research into the effects of yoga on BCRL, despite many women with BCRL stating that they are already using yoga along with conventional lymphoedema treatment [29]. However, yoga therapy as part of a holistic treatment for lower limb lymphoedema in India, has resulted in decreased levels of lymphoedema, and fewer infections. It focuses on lymphatic clearing by using specific postures, deep diaphragmatic breathing, elevation of legs and relaxation [30].

Yoga can offer a holistic approach to the management of BCRL as the practices promote progressive physical postures (*asana*) with breath awareness, breathing exercises (*pranayama*), meditation and relaxation. Research into the efficacy of yoga for women during and after breast cancer treatment (without lymphoedema) reports improved quality of life [31]and immunity [32,33] as well as reduction of pain [34] and fatigue [35]. Other improvements have been reported in flexibility [36], grip strength, shoulder function [37] and body image [38]. These outcomes may be transferrable to women with BCRL.

The aim of this study is to evaluate the effects of an eight-week integrated yoga intervention on women with BCRL. The primary objectives are to evaluate the effect of regular yoga participation on lymphoedema (amount of fluid and tissue density), and its associated symptoms (sensations, pain, fatigue) and to assess its impact on quality of life. Secondary objectives are to determine the effect of yoga on the upper body, in particular shoulder mobility and strength and mobility of the thoracic spine. A further objective is to investigate participant perceptions about the effectiveness of the yoga intervention. Information about daily and weekly physical activity as well as demographic information will also be collected.

We hypothesise that an eight-week integrated yoga intervention will have a beneficial effect on BCRL as indicated by reduced swelling of the affected arm, reduced tissue density, fewer symptoms of pain, fatigue and sensations and their limiting effects, improved quality of life and upper body functioning.

## Methods/design

### Study design and setting

This study is designed to be a randomised controlled trial for women with stage one BCRL, as defined by the International Society of Lymphology [16]. The study will compare the results of a group who receive an integrated yoga intervention, to a control group who will continue to follow current best-practice in self-management of lymphoedema from a manual given to all participants pre-randomisation, but who will receive no yoga intervention. The control group will be wait-listed for the yoga intervention and so will be invited to participate at the completion of the study.

Measurements will be conducted at baseline (week 0), after which participants will be randomised to the yoga intervention or the control group. Further measurements will be made at week 4 (mid-point), week 8 (on completion of the yoga) and at week 12 (four weeks after the intervention).

The trial will be held at two locations, Hobart and Launceston. Both locations will consist of an intervention and control group. The study will be conducted at Community Health Centres in both locations.

### Ethical considerations

The study has been approved by the University of Tasmania Social Sciences Ethics committee. Yearly progress reports will be made to the ethics committee and they will be notified of any adverse events promptly. The trial has been registered with the Australian New Zealand Clinical Trials Registry (ACTRN12611000202965, http://www.anzctr.org.au/default.aspx).

All women in the trial will receive information in a manual specifically developed for this trial on best current practice for management of secondary lymphoedema, based on the guidelines of the Australasian Lymphology Association and Lymphoedema Framework [16,39].

Women will be advised to continue with their usual treatment plan during the trial and to seek medical help in the case of infection or a flare-up of lymphoedema. Should this occur, women will be advised they can continue with the trial but told their measurements will not be included in the results, as it will change their status with regard to the eligibility criteria.

### Withdrawal from study

Participants will be able to withdraw from the trial at any time without prejudice, as set out in the information sheet distributed at the time of consent.

### Identification of eligible participants

Recruitment will occur over a two-month period throughout Tasmania. Key people, including professionals in the field of lymphoedema and relevant organisations such as the Cancer Council and Breast Screen, and support and exercise groups such as Encore and Dragons Abreast, will be contacted and asked to disseminate information about the trial to potential participants, through flyers and posters.

Media exposure will be sought, including articles in local newspapers and interviews on local radio. Posters will be put in major and rural hospitals, Women’s Health Centres (Hobart, Launceston) and Community Centres.

Individuals interested in participating will be asked to contact the principal investigator (AL) who will outline the study, including the yoga intervention, the home-practice, the methods and dates of measurement, after ascertaining if they are suitable for participation as per the inclusion and exclusion criteria. Eligible participants will be sent an information sheet and informed consent form. After receiving the forms, they will be telephoned in order to discuss any questions or concerns about the trial before returning the signed consent form.

After the signed consent form is returned, participants will be sent the dates for the trial (measurements and intervention) as well as a questionnaire with demographic and medical information to be completed and brought to baseline measurement. At this time, the manual for current best-practice will also be sent, and followed by another phone call to check for clarity.

### Sample size calculation

An *a priori* sample size calculation based on clinically significant changes between groups of between 10-20% in primary outcome measures with standard deviations of between 12 and 15% of the mean, or within groups 10% difference with an SD of 20%, indicated that numbers of between 13 and 19 participants would be required per group. Consequently, we plan on recruiting 20 participants per group to allow for a small number of withdrawals.

### Participants

Eligibility criteria based on previous exercise trials for women with BCRL will be followed [26,40]. Criteria for inclusion are completion of treatment for breast cancer in terms of surgery, radiotherapy and chemotherapy at least 6 months previously; unilateral secondary lymphoedema stage one related to surgery for breast cancer, as confirmed by a registered lymphoedema therapist; good English comprehension in order to understand the written forms and oral instructions and be able to give informed consent; aged > 18 years.

Criteria for exclusion are conditions of primary lymphoedema, recurrent cancer and other symptoms including infection or cellulitis, which would affect the woman’s lymphoedema and her quality of life adversely; severe psychological illness, as the yoga intervention would need to be specific to the person’s psychological illness in order to improve it; pregnant women and women with pacemakers, as these conditions are contra-indicated for the use of bio-impedance spectroscopy – one of the measuring tools for this study [41]; current lymphoedema treatment other than self-management, as this would affect the results.

Women will also be asked to refrain from commencing any new physical activity during the term of the trial as this could affect results.

### Randomisation

An individual not associated with the trial will perform the randomisation based on a computer- generated random number system. Group notification will be in a sealed envelope given to women after completion of the baseline measurement.

### The yoga intervention

The yoga intervention will consist of a weekly 90-minute yoga class for eight weeks by a qualified, accredited and experienced yoga teacher. Participants will also be given a 45-minute DVD made especially for this trial, with a shorter version of the class, and instructed to perform it daily. They will be given a log, in order to record their home sessions and make relevant comments.

Yoga practices and instruction approaches will be based on the Satyananda yoga® style of teaching. The breathing and *pranayama,* physical postures, meditation and relaxation techniques have systemised practices that will be taught to ensure uniformity of practice for outcomes [42]. This style, with its gentle repetitions of physical movements followed by rests, and use of modifications, may be well-suited to yoga for BCRL. Options for modifications will allow an individualised approach based on comfort, needs and preferences, in both the class and the home-practice DVD. Practices will be progressive during the eight weeks of the intervention.

The yoga intervention proposed in this study will consist of slow and deep breathing, a series of moving yoga postures followed by rests, *pranayama*, meditation based on mindfulness practices, and deep relaxation with arm elevation. The practices will be chosen to promote lymphatic clearing, improved postural awareness of the thoracic and shoulder area, as well as stress reduction.

Each yoga session will have appropriate warm-up and cool-down, following the guidelines of exercise for women with BCRL. The temperature of the room will be controlled to between 19–22 degrees Celsius (suitable for Tasmania) to ensure it does not become too hot, which can overload the lymphatic system [16].

### Primary and secondary outcomes

#### Characteristics of the sample

Women will complete questionnaires on demographic information, current health, as well as medical history pertaining to breast cancer and its treatment, and lymphoedema, onset and treatment prior to measurement. The completed questionnaires will be brought in at baseline, collected and entered into a specifically designed trial database.

### Primary outcome measures

#### Lymphoedema

All measurements of lymphoedema will be conducted with the participant resting in a supine position, with any compression sleeve removed. Due to the effect on the calculation of lymphoedema, the dominant and affected arm will be noted, and weight and height measured on the same equipment each time to calculate BMI.

#### Extra-cellular fluid

Extra-cellular fluid will be measured according to a recognized protocol [41], using bio-impedance L-dex™ XCA (Bio-Impedimed, Queensland), with electrodes placed at anatomical landmarks at the wrist of each arm and right ankle. An increase in extra-cellular fluid is paralleled by a decrease in impedance from the low-frequency electrical current. The result is recorded as a ratio to the non-affected limb, taking into account arm dominance [43]. As lymphoedema increases, so does the ratio of impedance [44]. The result, calculated from software provided by Bio-Impedimed, is recorded as an l-dex reading and any reading equal or higher than ten is considered an indication of clinically manifest lymphoedema.

#### Volume and percentage volume of arm and hand lymphoedema

Circumferential readings will be based on the protocol of the Australasian Lymphology Association [45], modified so the woman is lying not sitting. Both arms will be marked at the metacarpophalangeal joint, ulnar styloid and 10, 20, 30, 40 centimetres (cm) from the styloid process, using a pen and set square on the medial and lateral aspect of the arm, then circumference measured at each point by a Job non-stretch tape, recorded in cm. Finger circumferences will be measured distal to the web space, and recorded in millimetres (mm). Volume of lymphoedema and percentage of lymphoedema in the arm and hand will be calculated using the truncated cone formula [46] from the addition of circumference readings, using software provided by Flinders Lymphoedema Clinic, South Australia (SA), which compares the affected to the non-affected arm. An increase in fluid volume and % fluid volume readings equates to an increase in level of lymphoedema. Fluid volume will be recorded as millilitres (ml) and % fluid volume of ml.

#### Density of fibrous tissue

Density of fibrous tissue will be measured by a digital tonometer, model 1383 (Bio-medical Engineering, Flinders Medical Centre, SA), validated especially for this population [47,48], with an established protocol [49]. Tonometer measurements will be taken on the forearm 10 cm from the cubital fossa and on the upper arm 10 cm up from the cubital fossa in the middle of the lymph territory. Additional measurements will be taken at the anterior trunk at mid-clavicular line between the second and third ribs and the posterior trunk between the acromion and the first thoracic rib in the subscapular fossa. The digital tonometer measures the resistance of the tissues to compression - that is, the amount of fibrotic induration (collagen build-up) in the superficial tissues at a given point [46,50]. Each measurement will be followed by a three-second pause before retesting. A higher score for the digital tonometer denotes a higher level of tissue density. The result will be recorded in mm and the average of three recorded.

#### Physical sensations associated with lymphoedema

Visual Analogue Scales (VAS) have been validated as an effective method of recording individual perceptions of subjective parameters such as pain [51], and adapted in lymphoedema trials to include sensations specific to lymphoedema [40,52].

A VAS scale that has been developed for this trial will be used to measure the severity of lymphoedema sensations which each woman will define, such as heaviness, tingling, aching. The VAS scale will also measure pain and fatigue, and the degree to which sensations, pain and fatigue have limited her activity for that day. The VAS scale will record what each woman felt on the day of measurement. The scale is scored as 0 cm being ‘no discomfort’ and 10 cm being ‘the worst imaginable’.

#### Quality of life

A validated questionnaire [15], developed specifically to measure quality of life for people with lymphoedema (LYMQOL), will be used. Its upper limb version will be used in this trial. Scores are recorded for the day of measurement. Total quality of life score is recorded between 0–10, ten being the best and zero the worst. Its result is recorded as the number the woman recorded from 1 to 10. Independent scales for function, symptoms, appearance, emotions, are also included in the questionnaire. Each of these includes several questions which are marked from 1 to 4, four being the worst. The sum of answers for each area of function, symptoms, appearance and emotions are added, then divided by the number of questions in that section to give a score for each parameter. A higher score denotes a lower quality of life associated with that parameter. In this way, different aspects of quality of life can be recorded numerically and compared between intervention and control groups.

### Secondary outcomes measures

#### Range of motion of shoulder

The range of motion (ROM) of the shoulder of the non-affected, followed by the affected, arm will be measured using a two-armed goniometer, which has been validated for clinical trials [53] and used in trials for women with BCRL [54,55]. An established protocol [56] will be followed . Women will sit in a low-back chair with stable shoulder blades, and suitable back support, their knees bent to 90° and with their feet at hip width and flat on the floor. *Flexionabduction* and *extension* of the shoulder in the sagittal or coronal planes will be measured from the starting point of arms in anatomical position. *Interna*l and *external rotation* of the shoulder will be measured from the starting position of the arm abducted to 90°, forearm pronated and parallel to floor, palm down, with elbow bent to 90°. To prevent fatigue, the testing will be conducted in the following order: *flexi*o*ninternal rotationextensionabductionexternal rotation*. Endpoint of measurement will be full range, compensatory movements of the shoulder or trunk occurring, or women experiencing pain or tightness. The final result will be recorded in degrees as the best of three attempts. The higher the score the greater the range of motion.

#### Strength of shoulder

Muscle strength will be assessed using a Commander Powertrack II Muscle Tester (JTechMedical, Salt Lake City, Utah, USA), validated in a healthy population [57] and used in lymphoedema trials [7,58] following an established protocol for muscle testing [59]. The participant will sit in a stable position and the non-affected, then the affected, arm measured in turn, three times for each test. The arm will be raised to 90° for measurement of strength for *flexionhorizontal adduction* and *abduction.* The arm will be positioned slightly across the body for measurement of strength for *pectoralis major* and the arm elevated to 120° for strength of *serratus anterior*. To measure the strength of *extension,* the participant’s arm will be by her side. *Pectoralis minor* strength will be assessed with the participant in supine position. This is also the order of testing to prevent fatigue.

The strength of the arm or shoulder will be measured from the force applied against the resisted hand-held dynamometer, held by the assessor for a count of three seconds. Measurement will cease when full strength is applied, compensatory movements of the shoulder or trunk occur or pain is experienced. The best of three attempts will be recorded in Newtons (n). A high score denotes a stronger muscle action.

#### Grip strength

Hand grip strength will be assessed by using a hand-held grip dynamometer (Smedleys, TTM, http://www.stoeltingco.com), which has been validated for use in a clinical setting [60], and using a protocol common to lymphoedema trials [58]. The participant will sit in a stable position with her elbow bent at 90° and close to their body, palm facing inwards. Grip strength will be assessed by applying pressure to the dynamometer with the non-affected, followed by the affected, hand. Measurement ceases at full strength or if pain or instability occurs. The best of three attempts will be recorded in kilograms (kg). A high score denotes stronger grip strength.

#### Thoracic spine mobility

Thoracic spine mobility will be measured dynamically using video analysis in order to quantify the functional mobility of the spine during *flexion/extension, lateral flexion* and *rotation* following a validated protocol. [61]. Range of motion will be recorded by a video camera with backlighting, utilising reflective surface markers. Reflective markers will be placed on women’s skin at the following locations: left and right superior posterior iliac spines (LPSI, RPSI), spinal processes (S1, L3, L1, T6, T1) and left and right acromion (LACR, RACR). Reflective markers will also be placed on the wall behind, or on the floor around the chair in the case of rotation, to provide calibration references. At the initial assessment, the placement of markers will be measured and recorded on the participant’s data sheet, to ensure consistency in the placement of markers at each measurement.

Women will be tested standing in stable anatomical position for *flexion/extension* and *lateral flexion* with the camera at a distance of two metres, and in stable sitting position for *rotation* with the camera one metre overhead. The test will be performed three times with each movement stopping at the point of full range, instability or pain.

Video footage will be downloaded at the end of each measurement. Video data will then be analysed using Quintic™ sports biomechanics video analysis software 9.03 version 14 (http://www.quintic.com). Measures will be calculated from resting position to range in each direction and full range, and will be recorded in degrees from software specifically made for this trial (Human Life Sciences, UTAS), following the calculated measures described in another trial [61]. A higher score denotes greater movement. After analysis by Quintic, data will be entered into a specifically designed trial database.

As the use of video analysis with surface markers applied to the skin may be invasive for women who have been treated for breast cancer, women will have a choice of participation in this test.

#### Physical activity

Physical activity will be recorded at each session as it may impact other outcomes such as lymphoedema, sensation, pain, fatigue and arm movement. Physical activity in the week prior to each measurement period will be reported using the International Physical Activity Questionnaire (IPAQ-2005) short form, proven reliable in the Physical Activity for Lymphoedema (PAL) trial [62]. The questionnaire measures the amount of vigorous, moderate and walking activity in intensity and duration, as well as the number of minutes in sitting. The total time in each activity is multiplied by an intensity value to calculate total weekly activity reported in MET.min^-1^. The total MET.min^-1^ score will also be given a value of 1 = light; 2 = moderate; 3 = high. Sitting time is recorded as a total number of minutes for that week.

Physical activity for that day will be recorded on the VAS scale as a number from 0-10 with 0 indicating no activity and 10 indicating constant activity.

### Interview for yoga intervention women

A 20-minute audio-taped interview will be held at week 8 for the yoga intervention group and will be carried out by an independent assessor. Questions will be open-ended, in relation to the yoga sessions and DVD. Recorded interviews will be transcribed in full. An iterative-thematic approach will be used to analyse data by two researchers independently, then results compared and further analysis completed, by reporting data in themes and sub-themes according to frequency.

### Study measures at baseline and weeks 4, 8 and 12

All staff involved in measurements will be trained prior to commencement of baseline testing and use standardised protocol and validated instruments. Two qualified lymphoedema therapists will take the lymphoedema measurements, one in Hobart, one in Launceston. Other staff will be the same at both locations. All assessors will be blinded to group allocation and to previous results. Each measurement will be performed by the same assessor at each time-point.

Participants will be advised to abstain from alcohol 12 hours prior to testing, caffeine and heavy exercise two hours before and be allocated a time that will not change during the trial to ensure consistency of readings for lymphoedema levels.

Measurements for the thoracic spine sub-group will be held the day after the other measurements at baseline, weeks 8 and 12.

All equipment will be calibrated prior to each measurement period.

All data will be recorded onto a case record form for each woman then transcribed into a specifically designed trial database.

**Figure 1:**
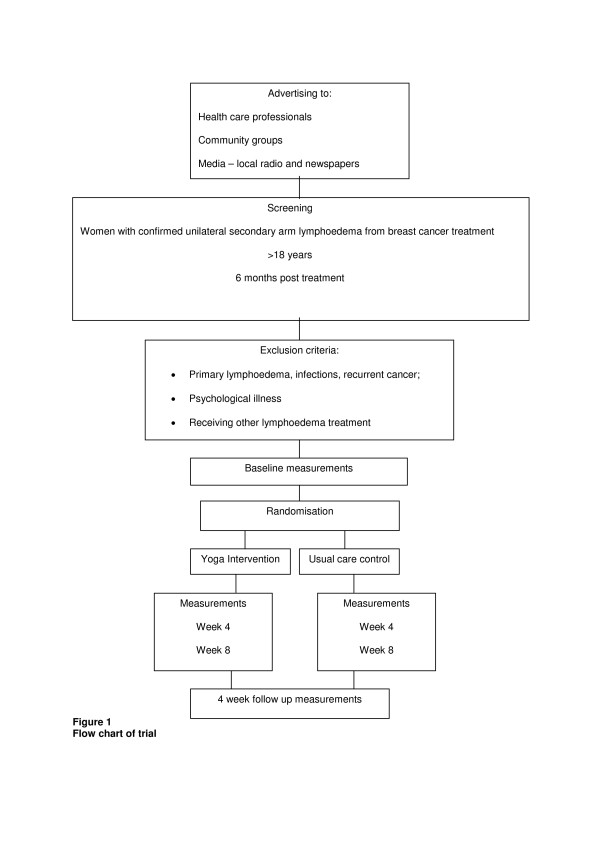
Flow chart of trial.

The study flow is summarized in Figure 1 and the study measurements in Table 1.

**Table 1:** Outcome measurements at baseline, weeks 4, 8, 12

**Baseline week 0**	**Week 4**	**Week 8**	**Week 12**
**measurements**	**Measurements-after 4 weeks intervention**	**Measurements-at completion intervention**	**Follow-up measurements**
Collection of medical, demographic questionnaires		Intervention group: interviews	
Log of daily yoga practice to yoga group	Collection of daily yoga log	Collection of daily yoga log	
Lymph measures:	Lymph measures:	Lymph measures:	Lymph measures:
· Bio-impedance	· Bio-impedance	· Bio-impedance	· Bio-impedance
· Circumference	· Circumference	· Circumference	· Circumference
· Tonometer	· Tonometer	· Tonometer	· Tonometer
· VAS symptoms	· VAS symptoms	· VAS symptoms	· VAS symptoms
Weight/height BMI	Weight/BMI	Weight/BMI	Weight/BMI
Arm measures:	Arm measures:	Arm measures:	Arm measures:
· Arm strength	· Arm strength	· Arm strength	· Arm strength
· Shoulder ROM	· Shoulder ROM	· Shoulder ROM	· Shoulder ROM
QOL:	QOL:	QOL:	QOL:
LYMQOL questionnaire	LYMQOL questionnaire	LYMQOL questionnaire	LYMQOL questionnaire
Physical activity:	Physical activity:	Physical activity:	Physical activity:
· IPAQ –short form	· IPAQ –short form	· IPAQ –short form	· IPAQ –short form
· VAS day’s activity	· VAS day’s activity	· VAS day’s activity	· VAS day’s activity
Thoracic spine – sub group:		Thoracic spine – sub group:	Thoracic spine – sub group:
· Spinal ROM video		· Spinal ROM video	· Spinal ROM video

### Measurements at weeks 4, 8, 12

Measurements and sequence of measurements will be identical to baseline.

### Compliance for attendance at yoga and home-practice DVD

A protocol will be put in place should a woman in the intervention have to miss a class, so that another class may be organised when possible. Attendance at the yoga class will be recorded weekly and DVD compliance self-recorded in a log, collected at weeks 4 and 8.

### Data analysis

Medical and demographic information will be analysed using descriptive analysis within SPSS (version 19SPSS Inc. IBM, USA 2010) to calculate means and standard deviations or percentage values. Differences between groups will be assessed using t-tests for comparing continuous variables and chi-square tests for categorical variables.

All statistical analyses for quantitative measurements will be performed using STATA statistical software (version 12; 1985-2011; STATA corp.; College Station; Texas, USA). Binary categorical data will be analysed for variability between the groups using logistic regression. Parametric longitudinal data will be analysed via mixed methods linear regression. Non-parametric data will use ordinal logistic regression. Post-hoc testing will be performed on both parametric and non-parametric data using the Holms test to locate the means that are significantly different. Statistical significance will be set at p < 0.05.

Qualitative data from interviews will be analysed using an iterative-thematic approach.

## Discussion and conclusion

The aim of this trial is to determine the effect of a yoga intervention on women with secondary arm lymphoedema as a result of breast cancer treatment by comparing the measurements of the intervention group with those of the control group, as well as to assess the perceived effects of yoga on the women in the intervention group by interview.

As no such trial has occurred up to this time, the results will assist in determining the effects of yoga on aspects relating to the lymphoedema, quality of life and upper body functioning for women with BCRL in the hope of finding another safe self-care option for women with BCRL. Finally, the methodology will inform future studies with larger numbers, as well as teaching guidelines for use by qualified yoga teachers.

## Competing interests

The authors declare they have no competing interests.

## Authors’ contributions

AL, TB and ADW are responsible for the design of this trial and the construction of the measurement protocol. AL will conduct the yoga intervention. NP is responsible for the design of the lymphoedema methodology. MAI assisted in design of the trial and advice for aspects of the yoga intervention. DV designed software for interpretation of opto-electronic data for the thoracic spine tests. All authors read and approved the final manuscript.

## Pre-publication history

The pre-publication history for this paper can be accessed here:

http://www.biomedcentral.com/1472-6882/12/66/prepub
